# A Rare Case of Hyperplastic Dental Follicle Presenting With an Unusually Large Radiolucent Area

**DOI:** 10.1155/crid/2879891

**Published:** 2026-07-07

**Authors:** Satoshi Otsuki, Yuki Kunisada, Koichi Kadoya, Chizuru Kobayashi, Keisuke Nakano, Toshiyuki Kawazu, Soichiro Ibaragi

**Affiliations:** ^1^ Department of Oral and Maxillofacial Surgery, Graduate School of Medicine, Dentistry and Pharmaceutical Sciences, Okayama University, Okayama, Japan, okayama-u.ac.jp; ^2^ Department of Oral Pathology and Medicine, Graduate School of Medicine, Dentistry and Pharmaceutical Sciences, Okayama University, Okayama, Japan, okayama-u.ac.jp; ^3^ Department of Oral and Maxillofacial Radiology, Graduate School of Medicine, Dentistry and Pharmaceutical Sciences, Okayama University, Okayama, Japan, okayama-u.ac.jp

**Keywords:** case report, hyperplastic dental follicle, large radiolucent lesion, odontogenic hamartoma, unerupted tooth

## Abstract

Hyperplastic dental follicle (HDF) is a rare odontogenic hamartomatous lesion typically associated with unerupted teeth in children and adolescents and is often found incidentally on radiographic imaging. HDF usually presents as a pericoronal radiolucency of 2–3 mm in diameter and is commonly difficult to distinguish from dentigerous cysts on radiographs. We report an unusual case of HDF in an 11‐year‐old male who was referred for evaluation of delayed eruption of the left maxillary canine. Panoramic radiography and cone‐beam computed tomography (CBCT) revealed a remarkably large radiolucent area, with a maximum dimension of 25.3 mm. The initial clinical diagnosis was a dentigerous cyst; however, incisional biopsy revealed solid tissue characteristics, and histopathological examination confirmed HDF, with no evidence of cystic lining, neoplastic transformation, or inflammation. Following the patient′s and parents′ preference for complete removal, resection of the lesion together with extraction of the impacted canine was performed under general anesthesia. At the 6‐month follow‐up, no recurrence was observed and bone regeneration was confirmed on radiographic examination. This case underscores the diagnostic challenge posed by atypical radiographic findings and highlights the necessity of histopathological confirmation. Given the exceptional size and potential for recurrence, long‐term follow‐up is warranted.

## 1. Introduction

Hyperplastic dental follicle (HDF) represents a benign, nonneoplastic proliferation of the pericoronal connective tissue surrounding impacted teeth [1]. Although not formally classified as a distinct entity, the WHO Classification of Head and Neck Tumours (4th edition) mentions that pericoronal central odontogenic fibroma (COF)–like lesions may be considered as HDF. The lesion predominantly occurs in the pediatric and adolescent age group, with reported cases ranging from 5 to 19 years of age and a male‐to‐female ratio of approximately 1.4:1. Radiographically, HDF presents as a well‐defined radiolucent zone surrounding the crown of an unerupted tooth. In radiographic terms, a pericoronal space within 2–3 mm is generally considered within normal limits; enlargement beyond this threshold raises suspicion for HDF [2, 3].

Because of its nonspecific presentation, HDF is often difficult to distinguish from dentigerous cysts based on imaging alone, particularly when calcification is absent. Multiple calcifying hyperplastic dental follicles (MCHDFs) involving several teeth with calcification have been well‐documented; however, isolated HDF affecting a single tooth and presenting with radiolucency exceeding 10 mm remains exceptionally rare. We report a case of HDF with an unusually large radiolucent area to discuss its clinical significance and diagnostic challenges.

## 2. Case Presentation

### 2.1. Patient Information

An 11‐year‐old boy was referred to our department from his primary dental clinic for further evaluation and treatment of a radiolucent lesion in the left maxillary canine region associated with delayed eruption of the canine. He had no significant past medical history. His family history was unremarkable, with no familial history of odontogenic lesions or jaw cysts.

### 2.2. Clinical Findings

Intraoral examination revealed normal overlying mucosa, but a buccolingual bony swelling was palpable in the left maxillary canine region (Figure 1A). No tenderness, erythema, or signs of infection were noted. The left maxillary first premolar appeared distally displaced.

**Figure 1:**
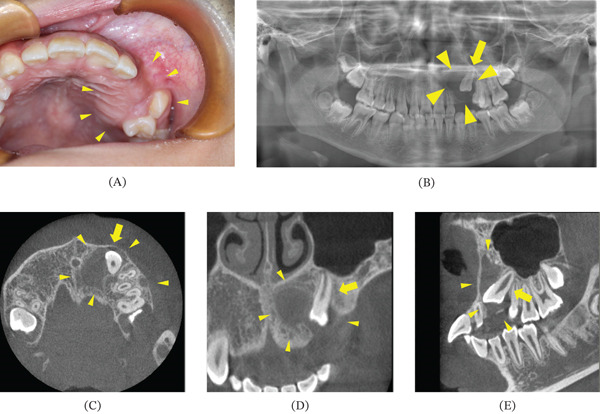
Clinical and radiographic findings at initial presentation. (A) Intraoral photograph showing normal overlying mucosa with buccolingual swelling (arrowhead). The left maxillary first premolar is distally displaced by the lesion. (B) Panoramic radiograph. (C–E) CBCT images demonstrating a large radiolucent area around the crown of the impacted left maxillary canine. Three‐dimensional CBCT measurements revealed a lesion size of 22.6 × 19.6 × 25.3 mm, with a follicular space of 14.6 × 6.5 × 8.7 mm along the tooth axis. The lesion shows a well‐defined border, an internally homogeneous pattern, and no calcifications, causing bone swelling, cortical thinning, and distal displacement of the left maxillary first premolar. (C) Axial view, (D) coronal view, and (E) sagittal view. The arrow indicates the impacted canine, and the arrowhead indicates the surrounding lesion.

### 2.3. Diagnostic Assessment

Panoramic radiography demonstrated a well‐defined radiolucent lesion surrounding the crown of the impacted left maxillary canine (Figure 1B). On cone‐beam computed tomography (CBCT) imaging, a well‐demarcated low‐attenuation area was observed in the left maxillary canine region, extending from the midline to beyond the left maxillary first premolar to the buccal aspect of the left maxillary first molar (Figure 1C–E). The internal structure of the lesion was homogeneous. Three‐dimensional measurements on CBCT revealed a lesion size of 22.6 × 19.6 × 25.3 mm, with the follicular space along the tooth axis measuring 14.6 × 6.5 × 8.7 mm (Figure 1C). Areas of cortical bone thinning were observed. No calcified material was detected within the lesion. The lesion also caused slight distal displacement of the left maxillary first premolar.

The differential diagnoses considered included: (1) dentigerous cyst, which was the most likely clinical diagnosis given the well‐defined pericoronal radiolucency associated with an unerupted tooth; however, incisional biopsy revealed solid tissue with no fluid content, effectively excluding a cystic structure; (2) odontogenic keratocyst (OKC), although the absence of scalloped margins, multilocular pattern, and the solid nature of the lesion made this unlikely; (3) unicystic ameloblastoma, which can occur in the pediatric population but was excluded based on the absence of cystic architecture; and (4) COF, which shares histological features with HDF but represents a true neoplasm rather than a hamartomatous proliferation [4].

Despite the unusually large size, HDF remained a plausible diagnosis because of its hamartomatous nature, the absence of neoplastic features on histopathological examination, and the characteristic dense collagenous tissue composition. Hamartomatous lesions are generally considered nonneoplastic proliferations and do not exhibit aggressive biological behavior.

Based on the clinical and imaging findings, a dentigerous cyst involving the left maxillary canine was suspected, and an incisional biopsy was performed. However, the lesion was found to be solid tissue rather than cystic, which was inconsistent with a dentigerous cyst. Histopathological examination confirmed a diagnosis of HDF, and odontogenic cysts and tumors were excluded.

### 2.4. Therapeutic Intervention

Based on the histopathological diagnosis confirming that the lesion was not a neoplastic disease, a treatment plan involving marsupialization followed by orthodontic traction was initially proposed to the patient and his parents. However, they declined orthodontic treatment or related procedures and requested complete removal of the lesion; therefore, surgical excision was performed.

Under general anesthesia, resection of the lesion together with extraction of the impacted left maxillary canine was performed. Where the lesion was in contact with the periosteum, separation from the surrounding bone was relatively easy, and the lesion was removed en bloc (Figure 2A,B).

**Figure 2:**
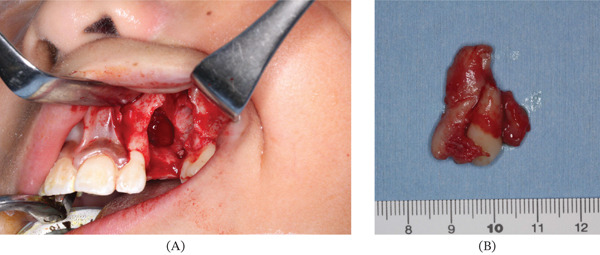
Intraoperative findings and resected specimen. (A) Intraoperative intraoral photograph showing the surgical field following mucoperiosteal flap elevation and bone removal to access the lesion. (B) The resected specimen, consisting of the lesion and the impacted canine, was easily separated from the surrounding tissue and excised en bloc.

### 2.5. Histopathological Findings

Histopathological examination of the resected specimen revealed that the lesion consisted predominantly of dense collagenous stroma with scattered fibroblasts, forming a solid mass (Figure 3A,B). Focal myxoid areas were observed within the stroma (Figure 3C). In the present case, odontogenic epithelium was not observed within the thickened fibrous tissue. This finding is consistent with previously reported HDF cases, in which epithelial remnants are often sparse or entirely absent [5]. Furthermore, the absence of odontogenic epithelium contrasts with COF, in which epithelial components are characteristically present within the tumor stroma [4]. No cystic epithelial lining, neoplastic cell proliferation, or inflammatory infiltration was observed (Figure 3A–C).

**Figure 3:**
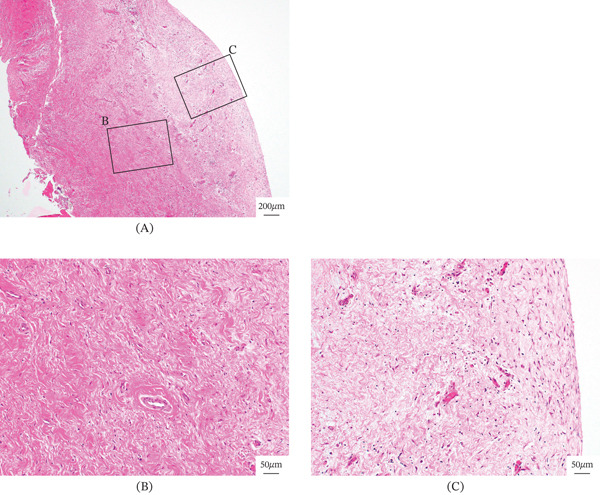
Histopathological findings (hematoxylin and eosin staining). (A) Low‐magnification view showing solid tissue composed predominantly of dense collagenous stroma. (B) High‐magnification view of the collagenous stroma showing scattered fibroblasts with no odontogenic epithelial rests identified. (C) High‐magnification view demonstrating focal myxoid change within the stroma. No neoplastic proliferation, inflammatory cell infiltration, or cystic epithelial lining was observed. Scale bars: A = 200 *μ*m; B,C = 50 *μ*m.

Immunohistochemical staining was not performed as the diagnosis was established based on characteristic histomorphological features on hematoxylin and eosin (H&E) staining. Based on these findings, odontogenic cysts and tumors were excluded, and a final diagnosis of HDF was established [3].

### 2.6. Follow‐Up and Outcomes

At the 6‐month postoperative follow‐up, the patient was clinically asymptomatic with no evidence of recurrence. Radiographic examination, including CBCT, demonstrated satisfactory bone regeneration at the surgical site (Figure 4A–C). No adverse events occurred during the follow‐up period, and the patient remains under observation for potential late recurrence. Given the unusually large size of the lesion and the limited literature on long‐term prognosis of isolated HDF, annual follow‐up with CBCT or panoramic radiography for a minimum of 3–5 years is planned.

**Figure 4:**
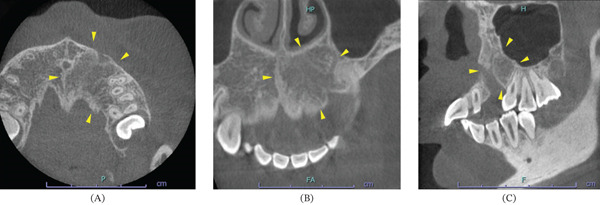
CBCT images at 6 months postoperatively. (A) Axial, (B) coronal, and (C) sagittal CBCT images obtained 6 months after surgery. Satisfactory bone regeneration is observed at the surgical site. The areas of newly formed bone are indicated by arrowheads.

### 2.7. Timeline

The clinical progression timeline for this patient, from initial referral through the 6‐month postoperative follow‐up, is illustrated in Figure 5.

**Figure 5:**
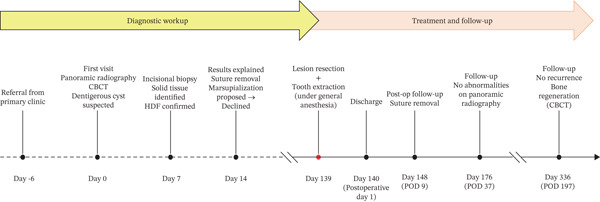
Clinical timeline. Flowchart illustrating the clinical timeline of the patient from initial referral through the 6‐month postoperative follow‐up, including diagnosis, treatment decision, surgical intervention, and follow‐up outcomes. Time interval not to scale.

## 3. Discussion

The radiographic appearance of HDF closely mimics that of a dentigerous cyst, presenting a significant diagnostic challenge when imaging alone is relied upon. In the majority of documented cases, the enlarged pericoronal space in HDF has been reported to remain within a few millimeters and typically displays a symmetric distribution around the crown. In the present case, the diagnosis of HDF was made based on the aforementioned histopathological findings. A condition with similar histologic features is MCHDFs, which involves calcifications at multiple sites and has been reported in numerous cases [6, 7]. In contrast, reports of isolated HDF confined to a single tooth are limited. Although in most published cases (including MCHDF), the radiolucent width does not exceed 5 mm, the lesion in our case measured 22.6 × 19.6 × 25.3 mm, with a follicular space of 14.6 × 6.5 × 8.7 mm along the tooth axis, far exceeding the typical dimensions reported in the literature [2, 3, 5–10] (Table 1).

**Table 1:** Summary of previously reported cases of hyperplastic dental follicle (HDF) and HDF‐like lesions.

Age	Sex	Site	Size of radiolucency	Clinical diagnosis	Final diagnosis	Year	Author
18	M	18	3–4 mm	Odontoma	HDF	1991	Fukuta et al. [5]
5	F	36 and 46	3–4 mm	Eruptive interdental mesenchymal calcifying hamartoma	HDF	2003	Onishi et al. [8]
17	M	17 ,27, and 36	5–6 mm	MCHDF	MCHDF	2010	Cho et al. [6]
11	M	17, 13, 23, 27, 37, 36, and 47	6–7 mm	MCHDF	MCHDF	2011	Cho et al. [6]
14	M	17, 13, 23, 27, 37, and 47	4–6 mm	MCHDF	MCHDF	2011	Cho et al. [6]
11	M	17, 27, 37, and 47	6–8 mm	MCHDF	MCHDF	2011	Cho et al. [6]
15	M	17, 15, 25, 27, 37, 35, and 47	3–5 mm	MCHDF	MCHDF	2011	Cho et al. [6]
19	M	18, 17, 27, 28, 38, 37, 33, 43, 47, and 48	3–4 mm	MCHDF	MCHDF	2013	Jamshidi et al. [7]
14	M	47	4 mm	Dentigerous cyst	HDF	2014	Schmitd et al. [2]
15	F	48	7–8 mm	Dentigerous cyst	HDF	2019	Gomes et al. [3]
11	F	33	4–5 mm	Postoperative odontoma	HDF	2022	Takahashi et al. [9]
16	M	15, 18, 24, 28, 34, 35, 37, 38, 43, 44, 45, 47, and 48	3–4 mm	MCHDF	MCHDF	2022	Rodrigues et al. [10]
14	F	36, 46, and 47	3–4 mm	MCHDF	MCHDF	2022	Rodrigues et al. [10]
11	M	23	10–12 mm	Dentigerous cyst	HDF	2024	This case

*Note:* Comparison of the width of the radiolucent zone: in most reported cases, the width is approximately 5 mm, whereas in the present case it exceeded 10 mm.

Abbreviation: MCHDF, multiple calcifying hyperplastic dental follicles.

Although the pathogenesis and clinical behavior of HDF remain incompletely understood, when the impacted tooth is favorably positioned and adequately supported, enucleation of the lesion or marsupialization with orthodontic traction has been reported to facilitate eruption of the permanent tooth. However, this approach may be less predictable in cases with extensive lesions and significant displacement of the tooth. In the present case, the lesion exceeded 25 mm in maximum dimension and was associated with cortical thinning and displacement of adjacent teeth, suggesting limited potential for successful orthodontic eruption. Furthermore, prolonged treatment duration and the risk of incomplete resolution were also considered.

The unusually large size observed in this case may be related to the hamartomatous growth potential of HDF and prolonged retention of the impacted tooth. The molecular mechanisms underlying such exceptional enlargement remain speculative; however, HDF is regarded as a hamartomatous proliferation rather than a true neoplasm, implying that its growth is driven by disorganized but nonautonomous tissue development [1, 3]. The prolonged retention of the impacted canine may have provided sustained local stimulation, contributing to progressive stromal expansion. The homogeneous internal structure and absence of calcification further support a purely fibrous hamartomatous process, distinguishing it from MCHDF [6, 7]. Considering that the contralateral canine had already erupted and its root formation was nearly complete at initial presentation, it is likely that the lesion had developed and progressed over a period of approximately 1–2 years.

Based on these considerations, marsupialization with orthodontic traction was initially proposed; however, the patient and his parents declined this option and opted for surgical excision. Although tooth extraction in a growing patient carries implications for occlusal development, the extent of the lesion, the degree of tooth displacement, and the patient′s preferences collectively supported surgical excision as a justifiable approach in this case. When an unerupted tooth is present, early detection, appropriate imaging evaluation, and individualized management based on these findings and patient preferences are warranted.

Although a neoplastic process was excluded histologically in this case, the unusually large size and the paucity of prior reports prompted us to plan long‐term follow‐up to monitor for any potential neoplastic change. Although the follow‐up period was limited to 6 months, no signs of recurrence were observed; however, long‐term follow‐up is necessary given the unusually large size of the lesion.

### 3.1. Strengths and Limitations

The strengths of this report include the detailed histopathological examination that enabled a definitive diagnosis, the comprehensive imaging evaluation using both panoramic radiography and CBCT, and the documentation of an exceptionally rare presentation of HDF with a radiolucent area exceeding 20 mm. To our knowledge, this represents one of the largest reported cases of isolated HDF confined to a single tooth. This report has several limitations. First, it is a single case report, and the findings may not be generalizable. Second, the follow‐up period of 6 months may be insufficient to fully assess the long‐term prognosis and potential for late recurrence. Third, the mechanisms underlying the unusually large size of this HDF remain unclear and warrant further investigation.

## 4. Conclusion

We report a rare case of HDF presenting with an unusually large radiolucent area exceeding 20 mm, which initially mimicked a dentigerous cyst. This case demonstrates that HDF should be included in the differential diagnosis of large pericoronal radiolucencies, even when the size exceeds the typical range. Histopathological examination remains essential for definitive diagnosis. Long‐term follow‐up is recommended given the exceptional size and limited understanding of the biological behavior of large HDF lesions.

## Author Contributions

Satoshi Otsuki.: writing – original draft, investigation (surgical treatment), and data curation; Yuki Kunisada.: conceptualization, writing – review and editing, investigation (surgical treatment), and project administration; Koichi Kadoya.: writing – review and editing and investigation (surgical treatment); Chizuru Kobayashi.: investigation (surgical treatment); Keisuke Nakano: investigation (histopathological analysis) and validation; Toshiyuki Kawazu.: investigation (radiological analysis) and validation; Soichiro Ibaragi.: supervision, writing – review and editing, and funding acquisition.

## Funding

No funding was received for this manuscript.

## Ethics Statement

This case report was conducted in accordance with the Declaration of Helsinki (1965) and its later amendments. This case report is not a research project; therefore, it does not have an IRB approval number. This report was written according to the CARE guidelines.

## Consent

Written informed consent was obtained from the patient′s parents for publication of this case report and accompanying images. The patient′s parents were informed of the clinical findings, differential diagnoses, and treatment options at each stage of management, and actively participated in all treatment decisions. Formal documentation of the patient′s perspective was not collected separately, as the patient was a minor. All personally identifiable information has been anonymized.

## Conflicts of Interest

The authors declare no conflicts of interest.

## Data Availability

Data sharing is not applicable to this article as no datasets were generated or analyzed during the current study.

## References

[1] Doğan Ö. , Yılmaz N. , and Hatipoğlu H. , Topal B. G. , Hyperplastic Dental Follicle, Lesions Associated With Unerupted or Erupting Teeth in Child and Adolescent Patients, 2025, BIDGE Publications, 58–75, 10.70269/KF68UPLFUIBO.

[2] Schmitd L. B. , Bravo-Calderon D. M. , Soares C. T. , and Oliveira D. T. , Hyperplastic Dental Follicle: A Case Report and Literature Review, Case Reports in Dentistry. (2014) 2014, 251892, 10.1155/2014/251892. 25374700 PMC4206926

[3] Gomes V. R. , Melo M. C. S. , Carnei H. C. , Pinho Filho J. E. , and Teixeira M. A. , Hyperplastic Dental Follicle: Case Report, Jornal Brasileiro de Patologia e Medicina Laboratorial. (2019) 55, no. 3, 315–320, 10.5935/1676-2444.20190027.

[4] Mosqueda-Taylor A. , Martinez-Mata G. , Carlos-Bregni R. , Vargas P. A. , Toral-Rizo V. , Cano-Valdéz A. M. , Palma-Guzmán J. M. , Carrasco-Daza D. , Luna-Ortiz K. , Ledesma-Montes C. , and de Almeida O. P. , Central Odontogenic Fibroma: New Findings and Report of a Multicentric Collaborative Study, Oral Surgery, Oral Medicine, Oral Pathology, Oral Radiology, and Endodontology. (2011) 112, no. 3, 349–358, 10.1016/j.tripleo.2011.03.021, 21684774. 21684774

[5] Fukuta Y. , Totsuka M. , Takeda Y. , and Yamamoto H. , Pathological Study of the Hyperplastic Dental Follicle, Journal of Nihon University School of Dentistry. (1991) 33, no. 3, 166–173, 10.2334/josnusd1959.33.166. 1748887

[6] Cho Y. A. , Yoon H. J. , Hong S. P. , Lee J. I. , and Hong S. D. , Multiple Calcifying Hyperplastic Dental Follicles: Comparison With Hyperplastic Dental Follicles, Journal of Oral Pathology & Medicine. (2011) 40, no. 3, 243–249, 10.1111/j.1600-0714.2010.00968.x, 21059105. 21059105

[7] Jamshidi S. , Zargaran M. , and Mohtasham N. , Multiple Calcifying Hyperplastic Dental Follicle (MCHDF): A Case Report, Journal of Dental Research, Dental Clinics, Dental Prospects. (2013) 7, no. 3, 174–176, 10.5681/joddd.2013.028, 24082990. 24082990 PMC3779378

[8] Onishi T. , Sakashita S. , Ogawa T. , and Ooshima T. , Histopathological Characteristics of Eruption Mesenchymal Calcified Hamartoma: Two Case Reports, Journal of Oral Pathology & Medicine. (2003) 32, no. 4, 246–249, 10.1111/j.1365-2842.2004.01357.x-i1, 12653866. 12653866

[9] Takahashi N. , Maekawa T. , Izumisawa M. , Mouri H. , Yamada H. , Satoh K. , and Tanaka R. , A Hyperplastic Dental Follicle That Arose During the Follow-Up Period After Odontoma Removal, Dental Radiology. (2022) 62, no. 1, 35–38, 10.11242/dentalradiology.62.35.

[10] Rodrigues L. G. , da Silva V. B. , Carmelo J. C. , Khouri M. S. , Mendes P. A. , and Manzi F. R. , An Imaging Perspective to Multiple Calcifying Hyperplastic Dental Follicles - A Report of Three Cases, Annals of Maxillofacial Surgery. (2022) 12, no. 2, 227–230, 10.4103/ams.ams_130_22, 36874791. 36874791 PMC9976857

